# Imeglimin amplifies glucose-stimulated insulin release from diabetic islets via a distinct mechanism of action

**DOI:** 10.1371/journal.pone.0241651

**Published:** 2021-02-19

**Authors:** Sophie Hallakou-Bozec, Micheline Kergoat, Pascale Fouqueray, Sébastien Bolze, David E. Moller

**Affiliations:** 1 Poxel SA, Lyon, France; 2 Metabrain Research, Maisons-Alfort, France; Tohoku University, JAPAN

## Abstract

Pancreatic islet β-cell dysfunction is characterized by defective glucose-stimulated insulin secretion (GSIS) and is a predominant component of the pathophysiology of diabetes. Imeglimin, a novel first-in-class small molecule tetrahydrotriazine drug candidate, improves glycemia and GSIS in preclinical models and clinical trials in patients with Type 2 diabetes; however, the mechanism by which it restores β-cell function is unknown. Here, we show that imeglimin acutely and directly amplifies GSIS in islets isolated from rodents with Type 2 diabetes via a mode of action that is distinct from other known therapeutic approaches. The underlying mechanism involves increases in the cellular nicotinamide adenine dinucleotide (NAD^+^) pool—potentially via the salvage pathway and induction of nicotinamide phosphoribosyltransferase (NAMPT) along with augmentation of glucose-induced ATP levels. Further, additional results suggest that NAD^+^ conversion to a second messenger, cyclic ADP ribose (cADPR), via ADP ribosyl cyclase/cADPR hydrolase (CD38) is required for imeglimin’s effects in islets, thus representing a potential link between increased NAD^+^ and enhanced glucose-induced Ca^2+^ mobilization which—in turn—is known to drive insulin granule exocytosis. Collectively, these findings implicate a novel mode of action for imeglimin that explains its ability to effectively restore—β-cell function and provides for a new approach to treat patients suffering from Type 2 diabetes.

## Introduction

Type 2 diabetes (T2DM) is characterized by insulin resistance plus β-cell dysfunction [1]. Existing therapies may only be partially effective or not well tolerated [1]. Glucagon-like peptide receptor (GLP1) agonists act on β-cells to amplify GSIS [2]. However, these agents are peptides with limited oral bioavailablity and are usually administered parenterally. Therefore, the pursuit of newer therapies, in particular small molecules which could function to reverse β-cell dysfunction, is warranted.

Imeglimin is a novel oral antidiabetic drug to treat Type 2 diabetes. Its novel structure and proposed mechanism of action establishes the first in a new tetrahydrotriazine class called the “glimins” [3]. Three Phase III clinical trials were recently completed and strong efficacy was seen in multiple trials [3–5]. Imeglimin’s mode of action involves dual effects; to ameliorate insulin resistance and potentiate GSIS [6, 7].

Imeglimin has prominent effects to reverse β-cell dysfunction and amplify GSIS: it ameliorates hyperglycemia in models with pancreatic deficient β-cell mass and function including neonatal streptozotocin (N0STZ) diabetic rats and Goto-Kakizaki (GK) rats and increases insulinogenic index during glucose tolerance tests [6]); *in vivo* GSIS is enhanced in both lean and high-fat fed rats [8]; increased GSIS was seen in hyperglycemic clamps in non-diabetic and N0STZ-diabetic rats [6]. In addition, a strictly glucose-dependent effect to enhance insulin secretion was seen with non-diabetic isolated rat islets [8]. Moreover, 7 day administration of imeglimin to Type 2 diabetes patients substantially amplified net GSIS as assessed by hyperglycemic clamp [9].

Given major effects on GSIS, we tested the hypothesis that imeglimin could acutely and directly impact β-cell dysfunction using islets isolated from Type 2 diabetes animal models (GK and N0STZ-diabetic rats). As an emerging therapeutic option for patients, it is also important to elucidate the mechanism of action. Thus, we conducted a series of studies using islets isolated from GK rats to define effects on pathways leading to GSIS amplification. GK rats are a non-obese Type 2 diabetes model of “isolated” β-cell dysfunction; many features resemble human disease including a loss of first phase insulin secretion, reduced β-cell mass, reduced islet insulin content, inflammation in islets, and impaired islet mitochondrial function [10]. Here, we determined that the mechanism of action of imeglimin was distinct vs. common antidiabetic therapies (metformin or sulphonylureas) and independent from mechanisms mediating the effects of other agents known to affect GSIS (GLP1 receptor agonists or phospholipase C pathway modulators). In contrast, imeglimin increases NAD^+^ levels in GK rat islets, potentially via the “salvage pathway” involving NAMPT and also increases cellular ATP content, suggesting an improvement in mitochondrial function. Further, we provide evidence suggesting a link, via CD38 and the generation of key NAD^+^ metabolites, between the increased NAD^+^ pool and enhanced intracellular Ca^2+^ mobilization. These findings implicate a novel mode of action for imeglimin that could be further leveraged to support the selection of appropriate patients and enhance its clinical utility or to develop improved agents in this new therapeutic class.

## Methods

### Animals, islet isolation, insulin secretion and intracellular Ca^2+^

Animal studies were conducted at Metabrain Research (Maisons-Alfort, France) according to European guidelines (2010/63/UE—ETS 123), for duly authorized projects by CNREEA (National Ethics Committee, APAFIS projects N°0709, 2796, 4027) and were also approved by Metabrain Ethics Committee. Rats were housed 4 per cage in controlled room (22°C; 12 hour light-dark cycle) with ad libitum access to water and normal chow diet (A113 for GK rats, A04 for Wistar and N0STZ rats; Scientific Animal Food and Engineering, AUGY–France). N0STZ rats were obtained by intravenous injection of streptozotocin (100 mg/kg) of rat pups (Charles River, Saint-Germain-Nuelles–France) as described [11]; 11–12 week-old rats with hyperglycemia and defective GSIS were used [12]. Male Wistar rats (11–14 week-old; Charles River) and male GK rats (14-week old; Metabrain Research) were also used.

Rats were anesthetized with i.p. sodium pentobarbital and sacrificed by decapitation. Islets were prepared by injection of collagenase (Sigma) into the pancreatic duct and surgical removal of the pancreas. The pancreas was digested for 9–11 min at 37°C, filtered and rinsed (Hank’s buffer solution containing BSA), and purified with a Ficoll gradient (Sigma) followed by several washes. For static incubations, islets were handpicked and distributed into 24 well plates; 9–16 wells per group with 6–12 islets per well, depending on the experiment. Islets were incubated for 20–30 min in Krebs Ringer Buffer (KRB) 0.2% BSA at 37°C, and under 95% O_2_/5% CO_2_, with and without test compounds in low (2.8 mM) or high (16.7 mM) glucose (DMSO 0.1% for all conditions) followed by removal of supernatant samples (stored at -20°C until insulin was measured). Insulin levels were measured with an Elisa kit Alpco 80-INSRTU-E01 or 80-INSRT-E01 based on a solid phase two-site enzyme immunoassay. It is a direct sandwich technique in which two monoclonal antibodies are directed against separate antigenic determinants on the insulin molecule. During incubation, insulin in the sample reacts with peroxidase-conjugated anti-insulin antibodies and anti-insulin antibodies bound to a microtitration well. The bound conjugate is detected by reaction with 3, 3’, 5, 5’-tetramethylbenzidine. The reaction is stopped by adding acid, to give a colorimetric endpoint that is read by a spectrophotometer. Selected test agents included imeglimin (Poxel SA), GLP1 (SIGMA, ref. G8147), metformin (Merck KGaA), an imidazoline [13] phospholipase C (PLC) pathway activator (BL11282, Metabrain Research) and a PLC inhibitor (U73122, SIGMA Ref. U6756).

For perifusions, islets were distributed (12 per well; 4 well-plates) in KRB containing 5.5 mM glucose and BSA (5 mg/ml) and maintained at 37°C under 95% O_2_/5% CO_2_. In selected studies, islets were loaded with Fura-2-AM (7.5 μM) (Thermofisher scientific, ref. F1201) added to buffer for 1 hr followed by three buffer exchanges. Batches of 8 islets each were plated on a polylysine coated glass disposed in small perifusion chamber and perifused at 1 ml/min with Hepes-BSA (1mg/ml) buffer alternately containing glucose 2.8 mM or 16.7 mM with or without test compounds. Perifusate was collected every minute. For intracellular Ca^2+^, the chamber was placed on the stage of a NIKON TE300 microscope (37°C); individual islets were imaged via excitation at 340nm and 380nm and fluorescence detection (510nm) with a photomultiplier (Photon Technologies International, Princeton, NJ). Intracellular Ca^2+^ results were expressed as ratio of F340nm/F380nm. Insulin levels were measured via Elisa (Alpco 80-INSRTU-E01 or 80-INSRT-E01).

### Insulin secretion from human diabetic islets

5.000 IEQ (islet equivalents, a standard unit based on average islet diameter of 150μm) from a single human diabetic cadaveric pancreas were isolated by PRODO LABORATORIES (California–USA) and were provided by TEBU BIO (Le Perray-en-Yvelines, France). Upon receipt, islets were stabilized in culture in order to recover from transport. They were placed in a Prodo Labs islet specific media supplemented with antibiotic and glutamine/glutathione mixture overnight at 37°C, 95%O_2_/5% CO_2_. The following day, islets were dispersed in 24 well plates at the density of 40 IEQ/well in 1 ml of media (mixture of HAM’S F10 and DMEM) containing 2.8 mM glucose and placed overnight at 37°C and 95%O_2_/5% CO_2_. Prior to the insulin secretion test, islets were washed and incubated twice in 2.8 mM glucose media 1 hr at 37°C. For static incubation, islets were incubated for 30 min in media with 8 mM glucose to induce insulin secretion with or without added compound. The supernatant was collected after 30min. of incubation. Supernatants were kept at -20°C until an insulin assay was performed. Insulin levels were measured with an Elisa kit (Mercodia, ref. 10-1113-10).

### Measurement of intracellular analytes

For cAMP, GK islets were incubated 30 min in 2.8 mM glucose and then incubated 15 min in 2.8 or 16.7 mM glucose with or without test compounds plus a phosphodiesterase inhibitor (IBMX 1 mM) to prevent cAMP degradation. Supernatants were removed by centrifugation and islets were maintained at -80°C in lysis buffer (Amersham RPN225). cAMP levels were subsequently measured in a pool of 20 islets with an EIA kit (Amersham, ref. RPN225).

Dinucleotide content was determined with 20 islets/well in 96 well filter plates; islets were placed in KRB with 16.7 mM glucose with or without imeglimin or nicotinamide (Sigma). Gallotannin was also used where noted (Santa Cruz, K2613). After 20 min, supernatants were removed by centrifugation and islets were stored at -80°C followed by lysis in PBS-dodecyltrimethylammonium bromide solution; NAD^+^ and NADH were determined using the bioluminescent assay from Promega (G9071); NADP^+^ and NADPH were determined using the NADP/NADPH-GloTM assay Promega kit (G9081), a bioluminescent assay for detecting total oxidized and reduced Nicotinamide adenine dinucleotide phosphates.

For ATP and ADP, islets (50 per dish) were stabilized in 5 ml of KRB, 0.2% BSA with glucose 2.8 mM for 30 min followed by distribution into 24 well plates (20 islets/well) in KRB 0.2% BSA with glucose 16.7 mM with or without test compounds. After 10 min, islets were transferred to 96 well filter plates and then maintained at -80°C with ultrapure water. After lysis (ATP kit buffer), ATP content was measured by luminescence (ATP lite, Perkin Elmer, 6016643); ADP content was measured with a fluorimetric assay (Sigma Aldrich, ref. MAK033).

### NAMPT activity and gene expression

Islets were dispersed for a stabilization period of 60 min in petri dishes at the density of 50 islets per dish containing 5 ml of Krebs Ringer Buffer 0.2% BSA with 2.8 mM glucose. After this stabilization period, islets were handpicked at a density of 20 islets/well into 24 well-plates and placed in Krebs Ringer Buffer 0.2% BSA with glucose 16.7 mM with or without test compounds. After 20 min of static incubation, islets were kept at -80°C until intracellular NAMPT (iNAMPT) activity was measured. For iNAMPT determination, islets were lysed in 50 mM Tris-HCl pH 7.5/0.02% BSA, 0.1% Triton X-100; iNAMPT activity was determined in pools of 60 islets with a colorimetric Cyclex assay kit (Clinisciences, ref. CY-1251). Human recombinant (*E*. *coli*) NAMPT activity was measured using the same kit after 60 min incubation.

Frozen (-80°C) islets (pools of 20) were homogenized followed by extraction and purification (RNAzol kit). RT-PCR measurements employed the AMV reverse transcriptase system (Applied Biosystems 4368814) and Q-PCR reactions (7900HT Fast Real-Time PCR, Applied Biosystems) using primers corresponding to the NAMPT sequence (Table 1). Levels of NAMPT mRNA were expressed as increases or decreases in cycle time [Ct] numbers compared to control after normalization to β-actin housekeeping genes.

**Table 1:** RT-PCR primers used to measure NAMPT mRNA expression.

Gene	Sequence 5’ 3’	Bases	Tm	Accession number
**rbeta-actin forward**	**GGGAAATCGTGCGTGACATT**	**20**	**55**	**V01217j00691**
**rbeta-actin reverse**	**CAGGAAGGAAGGCTGGAAGA**	**20**	**53**	
**rNAMPT forward**	**CAGAAGCCGAGTTCAACATC**	**20**	**60**	**NM-177928**
**rNAMPT reverse**	**TTTCACGGCATTCAAAGTAGG**	**21**	**60**	

### CD38 knockdown in islets

Islets were cultured 24 hours in RPMI medium (11 mM glucose plus inactivated serum, antibiotics, glutamine, 10 mM HEPES) and then placed in 10 cm^2^ plates (100 islets, each), washed in PBS and incubated 15 min on ice in permeabilization buffer (Lyovec 40μl/100 islets/5ml medium, Invitrogen) with siRNA from Origen (10 nM scrambled sequence or 10 nM directed against CD38). Islets were then cultured for 48 hr before further testing; 15 to 20 wells per group (10 islets/well). Static incubation in 16.7 mM glucose with or without test compounds was followed by removal of supernatant samples for insulin measurements and transfer of islets tubes for RNA extraction as above; CD38 mRNA levels were measured as described above for NAMPT.

### Modulation of cADPR and NAADP signaling

Islets were distributed (50 per dish) in 5 mL RPMI medium (11 mM glucose), and cultured at 37°C in 95% O_2_ and 5% CO_2_ for 72 hr. For the last 17 hr, high concentration (200 μM) ryanodine (EnzoLife Sciences–Ref. ALX-630-062-M005), was added to selected dishes. After transfer to fresh dishes and incubation for 30 min (KRB/BSA buffer containing 2.8 mM glucose with or without ryanodine), islets were distributed (6 per well) in 24-well plates in KRB containing 16.7 mM glucose with and without the indicated stimuli or inhibitors that also included cADPR (1 mM; Biolog–Ref. C005-025), NAADP (50 nM; SIGMA N5655), or combinations of two agents. After 20 min. incubation, samples of supernatants were removed and stored at -20°C. Insulin levels were measured with an Elisa kit (Alpco 80-INSRTU-E01 or 80-INSRT-E01) based on a solid phase two-site enzyme immunoassay.

### Statistics

Statistical analyses were performed using a Kruskall-Wallis non parametric one way ANOVA test followed by the Dunn’s post test (GraphPad PRISM4). Where noted, comparison between two conditions was performed using an unpaired Student t-test. A p value of ≤ 0.05 was considered significant.

## Results

### Imeglimin amplifies GSIS in diseased rat islets

**Fig 1:**
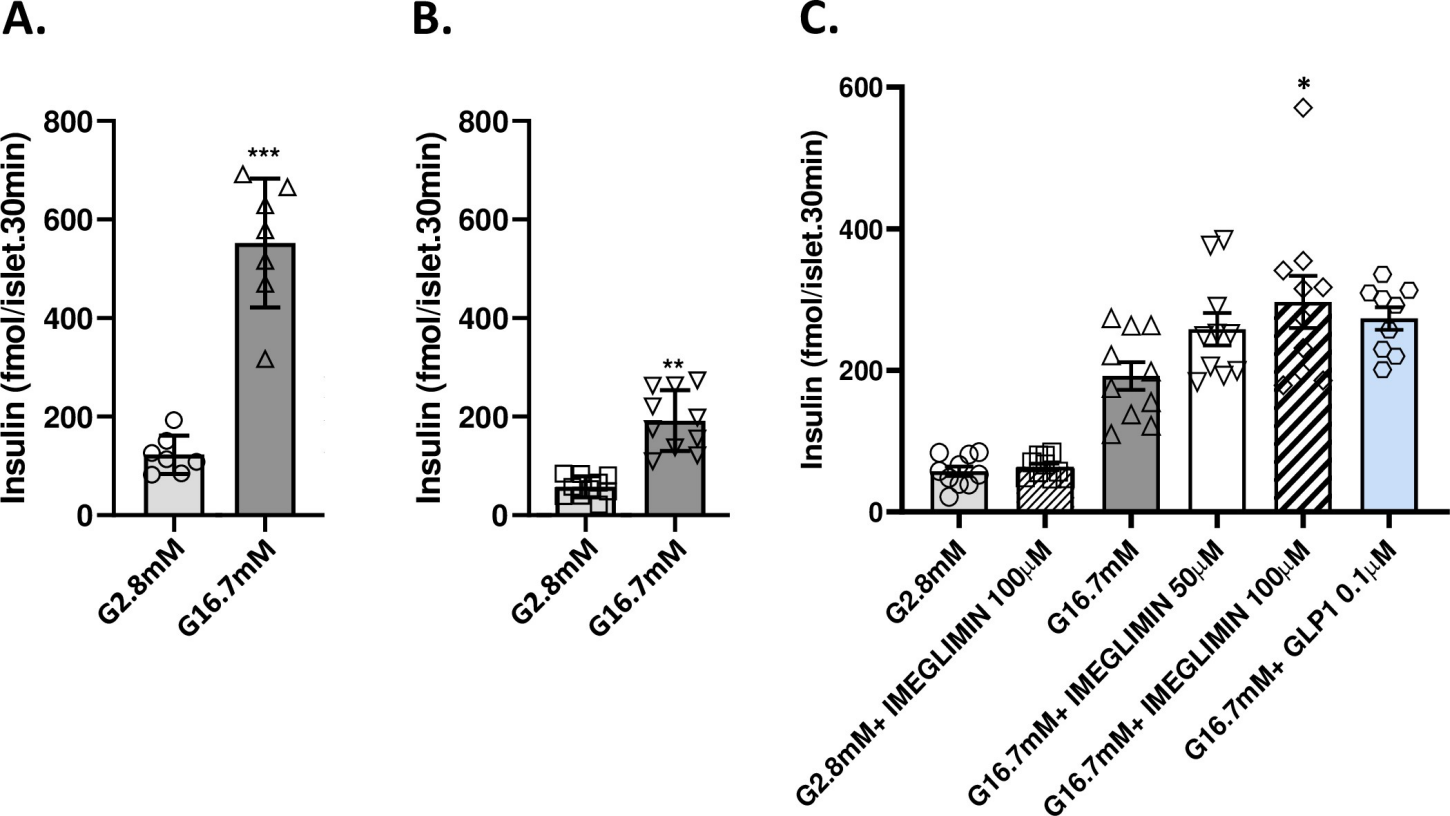
Imeglimin amplifies insulin secretion in islets from N0STZ rats. Wistar Rat Islets (A) vs. N0STZ Rat Islets (B). Islets from N0STZ or healthy Wistar rats were incubated in the presence of 2.8 mM or 16.7 mM glucose. Insulin levels were measured in supernatants after 30 min of incubation. **p<0.01, ***p<0.001 vs. respective low glucose values; mean ± SEM; n = 6 wells with 6 islets per well. Effect of imeglimin and GLP1 on Insulin Secretion from N0STZ Rat Islets (C). Islets from N0STZ rats were incubated in the presence of 2.8 mM or 16.7 mM glucose with or without the tested concentrations of imeglimin or GLP1 10^−7^ M. Insulin levels were measured in supernatants after 30 min of incubation. The effect of imeglimin at 100 μM was significant, *p<0.05, vs. high glucose alone; mean ± SEM; n = 9–10 wells with 6 islets per well (note that when using an unpaired Student t-test, GLP1 also achieved statistical significance, p = 0.0054).

β-cell function (GSIS) was impaired (-65% p<0.001) in N0STZ rat islets vs. Wistar control islets (Fig 1A and 1B). GLP1 induced a non-significant trend (+42%) towards increased GSIS in N0STZ islets (Fig 1C). In low glucose, imeglimin did not modify insulin secretion; in 16.7 mM glucose, increased insulin secretion was observed.

**Fig 2:**
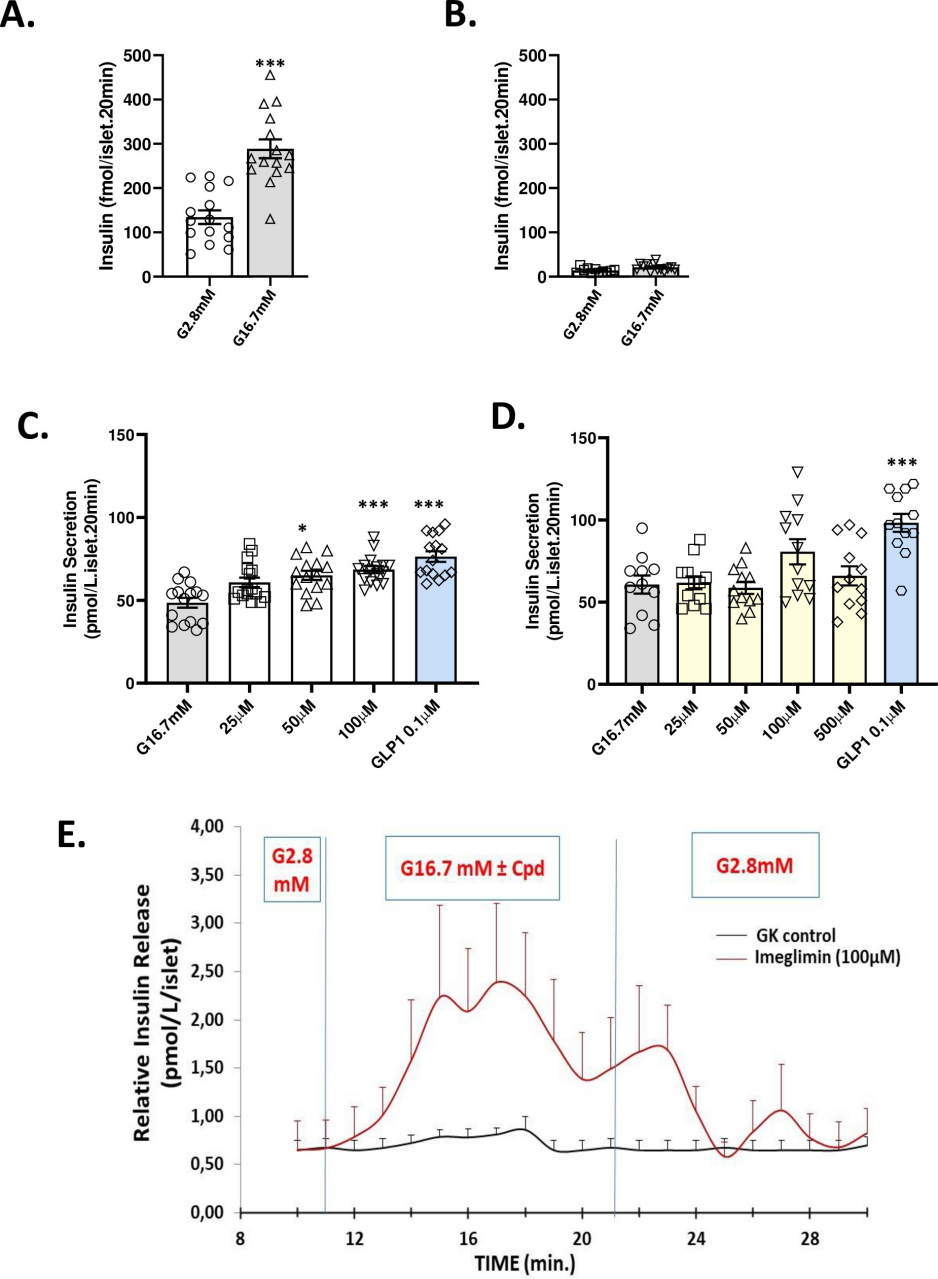
Imeglimin amplifies insulin secretion in islets from GK rats. Control Wistar Rat Islets (A) compared with GK Rat Islets (B). Islets from GK and Wistar rats were incubated in the presence of glucose 2.8 mM or 16.7 mM. Insulin levels were measured after 20 min of incubation. ***p<0.001 vs. respective control value; mean ± SEM; n = 6 wells with 6–10 islets per well. Imeglimin (but not Metformin) Amplifies Insulin Secretion from GK Rat Islets: Islets from GK rats were incubated in the presence of high (16.7 mM) glucose (grey bars) or with high glucose plus the indicated concentrations of imeglimin (C; open bars), metformin (D; yellow bars), or GLP1 as a control (blue bars; panels C and D). Significant increases in mean (± SEM) glucose-stimulated insulin release are noted vs. respective control values; *p<0.05, **p<0.01, ***p<0.001; n = 15 to 16 observations per group. Effects of imeglimin on Kinetics of Insulin Secretion from GK Rat Islets (E). Islets from GK rats were alternately perifused with 2.8 mM glucose for 10 minutes and 16.7 mM glucose with (red curve) or without (black curve) imeglimin (100μM) for 10 minutes (10 to 20 min) followed by perifusion with 2.8 mM for an additional 10 minutes. The insulin levels in the perifusate was measured every minute from 0 min to 30 min. Mean ± SEM insulin levels are shown (data are derived from 4 independent experiments for each group at each time point).

GSIS in GK rat islets was markedly impaired vs. a 2-fold response to high glucose in control Wistar islets (Fig 2A and 2B). Imeglimin potentiated GSIS; similar to the results obtained using N0STZ rat islets, imeglimin was without any effect at low glucose (S1 Fig). A dose-related effect was also evident with a magnitude similar to GLP1 (Fig 2C). Under the same experimental conditions, we confirmed that metformin could not enhance GSIS (Fig 2D). The effect of 100 μM imeglimin to ampifly insulin secretion in the presence of high glucose was replicated in 6 additional experiments (S1 Table). Using a perifusion system (Fig 2E), imeglimin was also shown to augment GSIS. In this context, the response to high glucose in control GK rat islets was negligible whereas islets from healthy Wistar rats were robustly responsive (S2 Fig). Imeglimin resulted in a partial restoration of GSIS relative to the response noted in Wistar rat islets (compare Fig 2E and S2 Fig).

In cadaveric islets derived from a single patient donor with Type 2 diabetes, we also observed an effect (+129%, p<0.05; n = 8–10) of imeglimin (100 μM) to amplify GSIS (S3 Fig).

### Imeglimin’s actions are distinct vs. other glucose-dependent mechanisms

**Fig 3:**
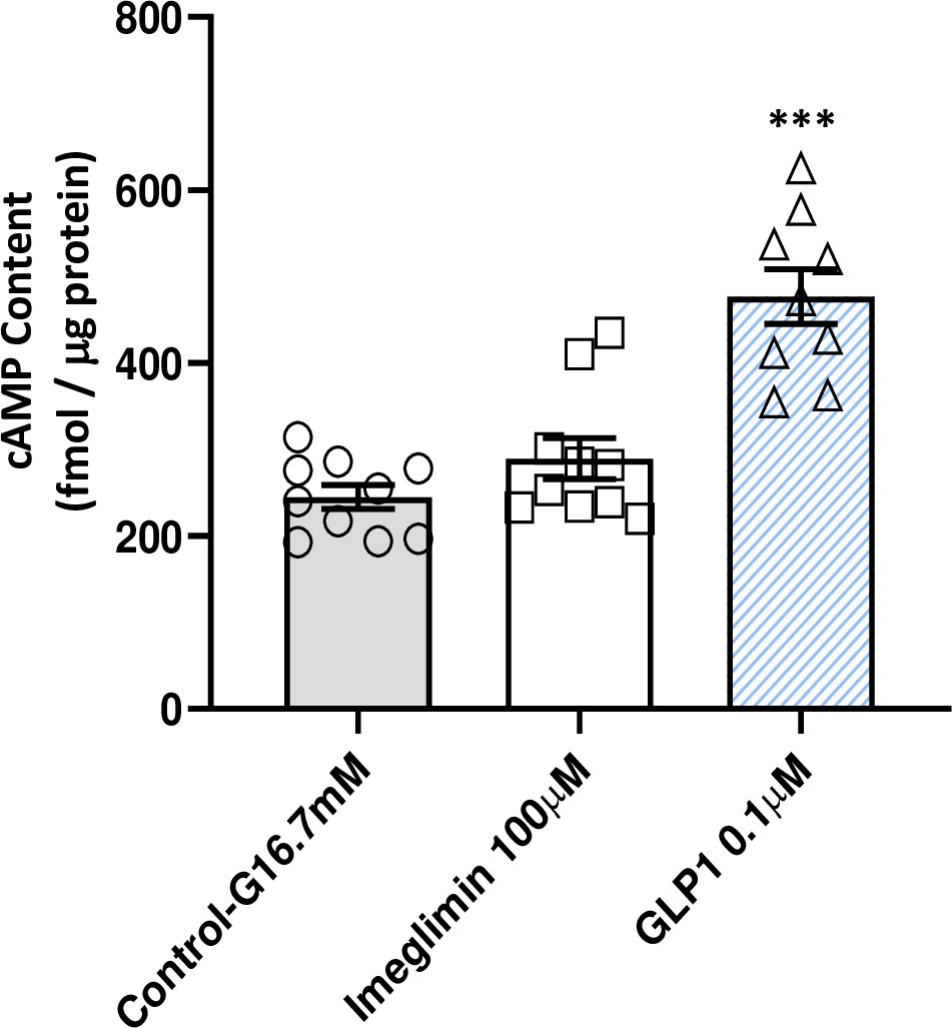
Imeglimin does not increase cAMP generation in isolated GK rat islets. In the presence of high glucose and the phosphodiesterase inhibitor IBMX, GLP1 (0.1μM) treatment increased the cAMP content of GK islets (+95%, ***p<0.001; n = 9). However, imeglimin (100 μM), produced no effect to increase cAMP under the same conditions. Mean ± SEM values are shown (n = 10). An additional independent experiment was also performed; levels of cAMP in each tested condition were not different between the two experiments.

The combination of imeglimin with GLP1 resulted in trends towards greater GSIS (S4 Fig). These results suggest that imeglimin and GLP1 may be acting via independent pathways to amplify insulin release. To confirm this hypothesis, we excluded an effect of imeglimin on cAMP, the classical mediator of GLP1 action, under the same conditions where GLP1 exerted a strong effect (Fig 3). In β-cells, phospholipase C (PLC) also mediates the potentiation of insulin secretion in response to molecules that include GPR40 (free fatty acid receptor 1) agonists that potentiate GSIS [14]. We excluded a role for PLC via use of a specific PLC inhibitor [15] (S5 Fig). These results suggest that imeglimin and GPR40 agonists act via independent pathways to amplify insulin release.

### Imeglimin modulates adenine dinucleotide and ATP levels

Adenine dinucleotides are known to modulate insulin secretion; we found that both imeglimin and exogenous nicotinamide induced increases in islet NAD^+^ content and the NAD/NADH ratio under high glucose conditions (Table 2).

**Table 2:** Imeglimin and nicotinamide effects on adenine dinucleotide and ATP, ADP content of GK rat islets.

	Control 16.7 mM Glucose	Imeglimin 25 μM	Imeglimin 100 μM	Nicotinamide 15 mM
NAD^+^	100 ± 5 100 ± 8	**155** **±** 18* **-**	123 ± 15 **131**± **11**^**#**^	**204** **±** 30*** 130 ± 14
NADH	100 ± 3 100 ± 1	111 ± 9 -	113 ± 13 100 ± 1	123 ± 12 105 ± 2
NAD/NADH	100 ± 5 100 ± 8	154 ± 26 -	**130** **±** **18**^**#**^ **131** **±** **9**^**#**^	**180** **±** 24*^#^ 124 ± 13
NADP^+^	100 ± 2 100 ± 4	109 ± 2 -	101 ± 3 114 ± 5	**116** **±** 5** **127** **±** 7**
NADPH	100 ± 0 100 ± 1	98 ± 1 -	94 ± 3 104 ± 2	94 ± 3 106 ± 3
NADP/NADPH	100 ± 1 100 ± 3	**110** **±** 2**	108 ± 3 109 ± 6	**125** **±** 4*** **121** ± 7*
ATP	100 ± 4 100 ± 11	**-**	**145** **±** 5*** 230 + 21*	**-**
ADP	100 ± 8 100 + 9	**-**	103 ± 8 102 + 9	**-**
ATP/ADP	100 ± 9 100 + 0	**-**	**142** **±** 10** 220 + 19*	**-**

Islets from GK rats were incubated in the presence of 16.7 mM glucose with or without imeglimin or Nicotinamide. Mean ± SEM values (n = 15 samples per group) are presented as the percentage of control. For measurements of NAD^+^, NADH, NADP^+^, and NADPH, mean values for each of two sets of experiments are shown separately (one with three experiments—3 batches of islets; a second with two experiments—2 batches of islets). ATP and ADP levels were determined in independent experiments with two separate batches of islets (n = 10 samples per group). Statistically significant results are noted in bolded text

*p<0.05

**p<0.01

***p<0.001. An unpaired Student t test was used for selected comparisons

#p<0.05

##p<0.01 vs. control.

As NAD^+^ is an essential co-factor for mitochondrial function [16], we also measured ATP levels. The measurement of islet ATP content was validated by assessing the acute (10 min.) effect of exposure to high (16.7 mM) vs. low (2.8 mM) glucose alone; a +47 + 10% increase in ATP was measurable in this context (p<0.05; n = 14–16 observations in each group). In the presence of high glucose, imeglimin significantly increased mean ATP content and the ATP/ADP ratio (Table 2). The effect of metformin was also characterized; no such effect was detected with metformin (S6 Fig). To confirm that increases in islet NAD^+^ are sufficient to amplify GSIS in diseased islets, we showed that insulin secretion and NAD^+^ content were increased by exogenous nicotinamide (S7 Fig).

### Increased NAD^+^ via the salvage pathway—increases in NAMPT expression and activity

**Fig 4:**
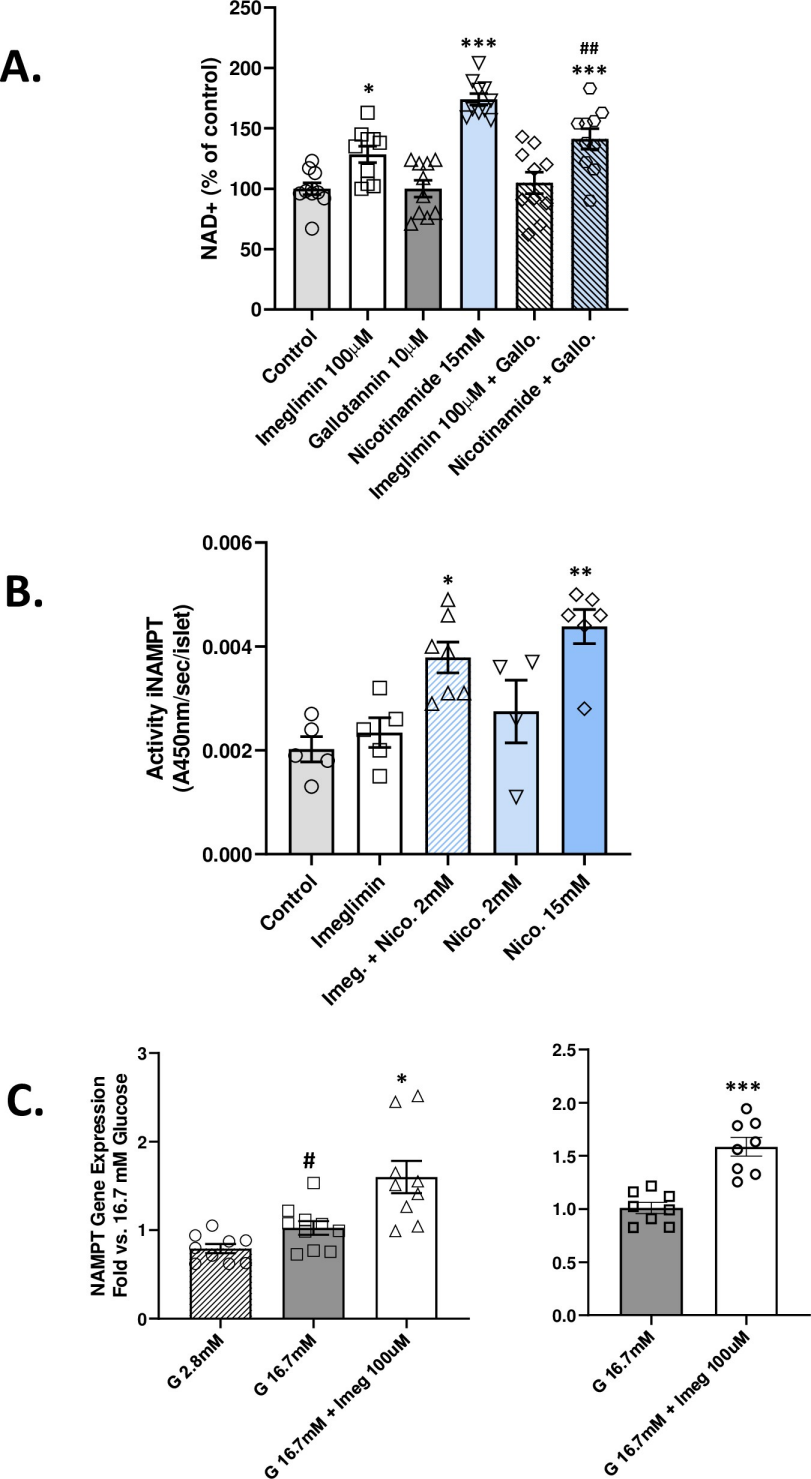
Imeglimin increases the NAD^+^ pool through increased synthesis. Gallotannin Effect on NAD^+^ (A). Islets from GK rats were incubated in the presence of 16.7 mM glucose with or without imeglimin (100 μM), or nicotinamide (15 mM); compounds were administered alone or in combination with gallotannin (10 μM). NAD^+^ was measured after 20 min incubation; mean (n = 10 in each group) ± SEM values are shown; *p<0.05, ***p<0.001 vs. Control; ## p<0.01 vs. nicotinamide alone. iNAMPT Activity (B). Islets from GK rats were incubated in the presence of 16.7 mM glucose with or without Imeglimin (100 μM), or nicotinamide (2 mM or 15 mM), or the combination of imeglimin and 2 mM nicotinamide. Intracellular (i) NAMPT activity was then measured; mean ± SEM (n = 5–6 per group) values are shown. *p<0.05, **p<0.01 vs. Control. In an independent experiment, iNAMPT activity was induced by the combination of imeglimin (100 μM) and 1 mM nicotinamide (+42%; p<0.05 vs. both control and nicotinamide alone). NAMPT mRNA Levels (C). Results from two separate experiments (Right and Left panels) are shown. NAMPT gene expression was determined by RT-PCR in islets from GK rats that were incubated for 30 min in the presence of 2.8 mM glucose (hatched bar), 16.7 mM glucose (solid bars) or 16.7 mM glucose plus imeglimin (100 μM; open bars). Mean (± SEM; n = 9–10 observations per group) levels of NAMPT mRNA are shown as fold vs. 16.7 mM glucose alone; #p<0.05 vs. 2.8 mM glucose; *p<0.05; ***p<0.001 vs. 16.7 mM glucose.

To assess if increases in the NAD^+^ pool are due to enhanced synthesis, we used Gallotannin, an inhibitor of nicotinamide mononucleotide adenylyl transferase (NMNAT), a key enzyme in the NAD^+^ synthetic pathway [17, 18]. Gallotannin (10μM) alone had no effect on NAD^+^. As expected, imeglimin or 15 mM nicotinamide increased NAD^+^ levels (Fig 4A). With Gallotannin co-administration, NAD^+^ content in imeglimin treated islets was no longer above control levels and NAD^+^ content in nicotinamide treated islets was partially suppressed. These results suggest that the effect of imeglimin on NAD^+^ content is mediated by increased synthesis.

At a low concentration (2 mM), the NAMPT substrate–nicotinamide—appeared to potentiate the effect of imeglimin on GSIS (+89% vs. +33% with imeglimin alone). Given this result, the activity of intracellular NAMPT, a key enzyme in the NAD^+^ salvage synthesis pathway, was assessed (Fig 4B). As expected, iNAMPT activity was greater with 15 mM nicotinamide (+117%, p<0.01) and not significantly increased at 2 mM. In the absence of NAMPT substrate (nicotinamide), imeglimin did not significantly modify iNAMPT activity; however, with 1–2 mM concentrations of nicotinamide, iNAMPT activity was significantly increased by the addition of imeglimin. Thus, in the presence of low concentrations of added substrate, imeglimin leads to increased NAMPT activity. The possible effect of imeglimin to directly modulate human recombinant NAMPT activity was also assessed. Recombinant NAMPT enzyme activity was not altered by imeglimin at six different concentrations (S8 Fig).

Since glucose rapidly induces NAMPT expression in isolated human islets [19]; the potential for imeglimin to upregulate NAMPT mRNA was interrogated. High glucose alone modestly induced NAMPT mRNA levels; added exposure to imeglimin further increased NAMPT mRNA (Fig 4C).

### Imeglimin’s effects are distinct vs. sulphonylureas

**Fig 5:**
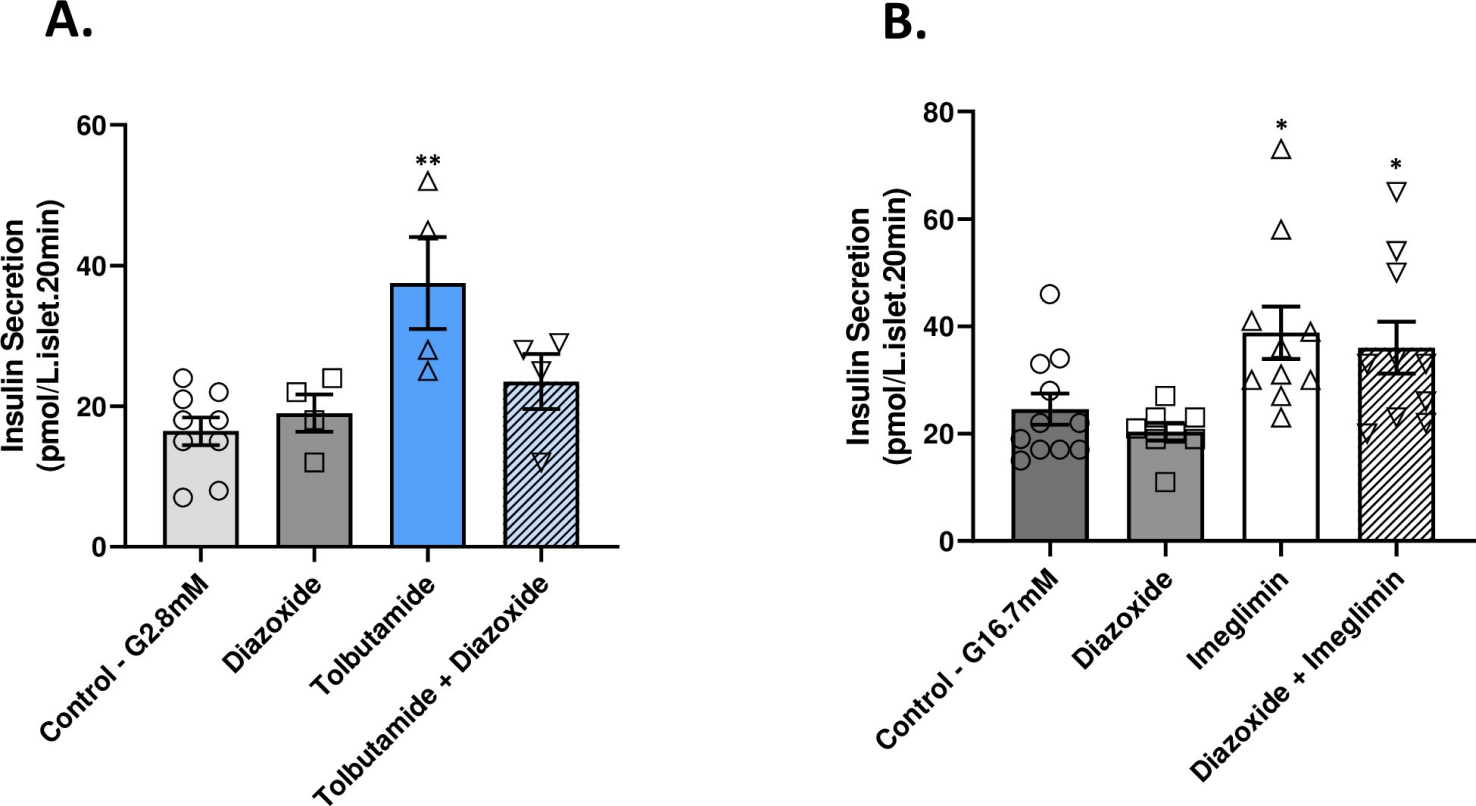
Imeglimin effect on insulin secretion is resistant to diazoxide. (A) Islets from GK rats were incubated in low (2.8 mM) glucose with or without diazoxide (400 μM), tolbutamide (500 μM), or a combination of both diazoxide and tolbutamide. (B) GK rat islets were incubated in high (16.7 mM) glucose with or without diazoxide (400 μM), imeglimin (100 μM), or a combination of both diazoxide and imeglimin. Samples were obtained after 20 min and subsequently assayed to determine insulin concentrations; *p<0.05, **p<0.01, vs. respective control value. Mean ± SEM values are shown.

Diazoxide opens K^+^-ATP channels to inhibit GSIS [20, 21]; sulphonylureas including tolbutamide mediate channel closure and glucose-independent insulin secretion [22]. As expected, tolbutamide (and glibenclamide) increased insulin secretion (Fig 5A; S9 Fig); diazoxide was also shown to inhibit the effect of tolbutamide (Fig 5A). Control experiments also showed that GK rat islets retain the ability to respond to KCl (S9 Fig). Imeglimin’s effect to augment GSIS was unaffected by diazoxide (Fig 5B). Taken together with the absence of an imeglimin effect on insulin secretion in low glucose, these results further suggest that imeglimin’s mode of action is distinct from sulphonylureas and may involve a pathway(s) that is independent of K^+^-ATP channels.

### Potential role of a CD38–cADPR-ryanodine receptor pathway in NAD^+^ mediated mobilization of intracellular Ca^2+^

**Fig 6:**
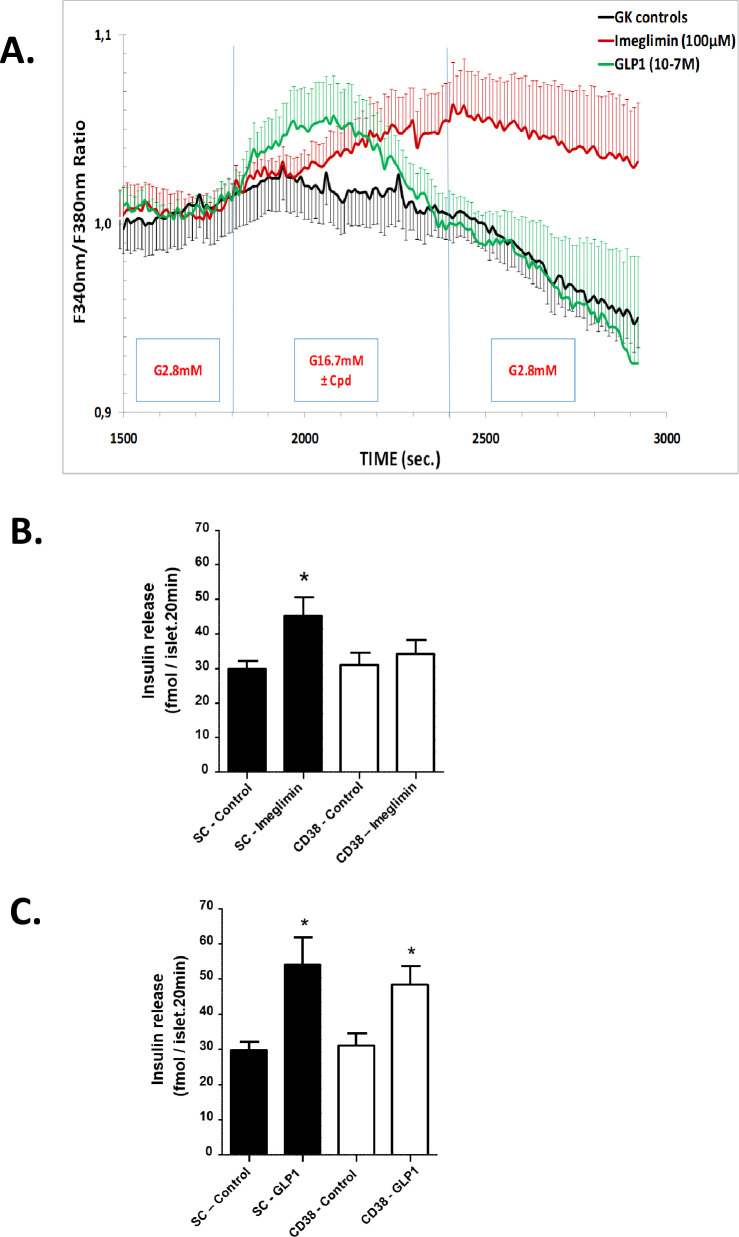
Potential role of CD38 and NAD^+^ metabolites to enhance insulin secretion via increasing intracellular Ca^2+^ in response to glucose. Measurement of Intracellular Ca^2+^ in Perifused GK Rat Islets (A). Islets from GK rats were perifused alternately with glucose 2.8 mM and 16.7 mM glucose without treatment for Controls (black curve), with imeglimin 100 μM (red curve) or with GLP1 0.1 μM (green curve) followed by a third period of perifusion with 2.8 mM glucose alone. Intracellular Ca^2+^ levels were measured from individual islets by successive excitation at 340 nm and 380 nm and detection of fluorescence emitted at 510 nm every 10 seconds. Results for each of the three groups (control, imeglimin, GLP1) are derived from 8 experiments with a total of 8 to 10 rats per group (8 rats for control and GLP1 groups, 10 for the imeglimin group). Insulin Secretion Response to Imeglimin and GLP1 With and Without CD38 Knockdown: Scrambled sequence siRNA control (SC-Control, solid bars) and CD38 siRNA (open bars) transfected GK rat islets were incubated for 20 min in high (16.7 mM) glucose with or without 100 μM imeglimin (B) or 0.1 μM GLP (C). Mean ± SEM (n = 15–20 per group) insulin release values are shown; *p<0.05 vs. respective control.

As expected, we also observed that imeglimin could induce increases in intracellular Ca^2+^ in response to glucose in GK islets (Fig 6A). This effect to induce an increase in intracellular Ca^2+^ was also not observed in conditions of continuous low glucose incubation (S11 Fig). We have also observed that glucose-induced Ca^2+^ mobilization in GK rat islets is impaired by more than 85% vs. Wistar rat islets studied in parallel in a perifusion assay (S10 Fig). Interestingly, the kinetics of imeglimin’s effect on intracellular Ca^2+^ (more sustained with a lag in returning towards baseline after switching to low glucose) appeared to differ from that observed with GLP1 (acute and transient). This difference is consistent with the notion that imeglimin and GLP1 operate via distinct pathways in augmenting GSIS. The pathway implicated in leading to increased intracellular Ca^2+^—via an increase in the cellular NAD^+^ pool (described below) might also be expected to result in a delayed return to baseline. Importantly, the lag in intracellular Ca^2+^ concentrations returning to baseline does not necessarily imply that imeglimin has glucose-independent effects to stimulate insulin secretion; indeed data shown in Fig 1C and S1 Fig show no effects on insulin secretion under low glucose conditions and insulin secretion returns to baseline within 3–5 minutes of switching to low glucose as shown in Fig 2E.

NAD^+^ is metabolized to cyclic ADP-ribose (cADPR) and nicotinic acid dinucleotide phosphate (NAADP) via CD38 (ADP ribosyl cyclase/cADPR hydrolase). Both metabolites are implicated in mobilizing internal Ca^2+^ stores, through activation of ER ryanodine receptors in the case of cADPR and via two-pore channels (TPCs) in the case of NAADP.

To assess the role of CD38, siRNA-mediated knockdown was employed. CD38 siRNA produced significant and reproducible decreases in CD38 mRNA (from -40% to -49%, p<0.01–0.05; S12 Fig) vs. control siRNA. When CD38 mRNA expression was only moderately reduced, imeglimin’s effect on GSIS was abolished (Fig 6B). In contrast, effects of GLP1 treatment were unaffected and there was no effect with scrambled (control) siRNA (Fig 6C). These results suggest that CD38 is required for the effect of imeglimin to potentiate GSIS.

Finally, we studied the effects of modulating signaling via cADPR or NAADP on insulin release (Table 3). GLP1 and imeglimin produced expected GSIS effects and exogenous cADPR (1.0 μM) also increased GSIS. cADPR’s effects to enhance Ca^2+^ mobilization (and GSIS) are reportedly mediated by ryanodine receptors (RyR) [23]; thus, high concentration ryanodine was used as a RyR inhibitor. In the presence of 200 μM ryanodine, the effects of either cADPR or imeglimin to augment GSIS appeared to be abrogated (Table 3). However, baseline glucose-stimulated insulin release was also modestly lower in the presence of 200 μM ryanodine vs. without ryanodine, thus complicating the interpretation of these data. Overall, these data suggest a role for cADPR in contributing to imeglimin’s effects to amplify glucose-stimulated Ca^2+^ mobilization and insulin secretion.

**Table 3:** Effects of modulating cADPRon glucose-stimulated insulin secretion.

	Insulin Secretion		
Treatment Group	pmol/L.islet.20 min	% of 16.7 mM Glucose Control	p value(s)
Control 16.7 mM Glucose	5.1 ± 0.8	100 ± 15	-
GLP1 (0.1 μM)	**14.1** **±** **1.5**	**274** **±** **29**	<0.001*
Imeglimin (100 μM)	**7.8** **±** **0.7**	**152** **±** **13**	<0.05*
cADPR (1.0 μM)	**7.3** **±** **0.5**	**143** **±** **10**	<0.05*
Ryanodine (200 μM)	3.2 ± 0.3	63 ± 6	NS
cADPR + Ryanodine	**4.3** **±** **0.4**	**84** **±** **8**	NS*; **<0.001#**
Imeglimin + Ryanodine	**5.4** **±** **0.8**	**105** **±** **15**	NS*; **<0.05#**

Islets from GK rats were incubated in the presence of 16.7 mM glucose for 20 min with or without the indicated compounds as shown; effects pathway inhibition (excess ryanodine)—with or without cADPR or imeglimin stimulation—are depicted in the lower portion of the table. Mean ± SEM values for insulin released (pmol/L.islet.20 min; also presented as % of 16.7 mM glucose control) are shown (n = 8–13 observations per group). Bolded values are statistically significant; p values vs. 16.7 mM glucose control (*) or vs. the respective single compound in combination treatments (#, first agent listed in Column one) are noted.

## Discussion

The prominant role of β-cell dysfunction in Type 2 diabetes is well established [24–28]. Here, we elucidated a novel mechanism by which imeglimin, a new potential anti-diabetic medication, improves β-cell function–an effect that has been clearly demonstrated *in vivo* in both animal models [6, 8] and humans [9].

Imeglimin ameliorates hyperglycemia in rodent models characterized by a primary β-cell defect–STZ-diabetic and GK rats [6]. Here, we determined that imeglimin could acutely and directly enhance GSIS (without any effect in low glucose conditions) with isolated islets from these models. Concentrations where imeglimin was effective (25–100 μM) are also aligned with human exposure levels (estimated ≈ 50 μM, unpublished; Poxel SA).

Several observations indicate that imeglimin’s mechanism is distinct vs. other therapeutic approaches. It is important to distinguish the effects of imeglimin from metformin since in liver there is an apparent overlap with respect to inhibition of gluconeogenesis and the potential to partially inhibit mitochondrial Complex I [6, 7]. We confirmed that metformin fails to directly potentiate GSIS, consistent with the literature [29, 30]; in addition, metformin had no effect on GK islet ATP (vs. significant increases with Imeglimin). GLP1 binding to its cognate G-protein coupled receptor induces rapid activation of plasma membrane associated adenylyl cyclase leading to clear increases in cAMP [2, 31]; imeglimin had no such effect. Sulphonylureas such as tolbutamide, are secretagogues in both low- and high-glucose; in contrast, the effects of imeglimin (like GLP1) are only glucose-dependent. We also found that, unlike sulphonylureas, imeglimin’s effect on GSIS was retained in the presence of diazoxide, a classical β-cell K^+^-ATP channel opener [32]. Together with the observed lack of effect on insulin secretion under low glucose conditions in this and prior [8] studies, these findings are consistent with the likelihood of a K^+^-ATP independent mechanism for imeglimin. Importantly, the GSIS enhancing effects of incretins like GLP1 also involve a diazoxide-resistant K^+^-ATP independent pathway [33]. GPR40 agonists and molecules in the imidazoline class have been pursued as GSIS enhancing therapies; these agents operate through PLC activation [14, 34] which was also excluded a requirement for imeglimin’s action.

Mitochondrial dysfunction is a key feature of β-cell dysfunction [35–37]; decreases in ATP generation have been described in islets from GK rats and patients with Type 2 diabetes [35, 38–40]. We previously showed that imeglimin can modulate mitochondrial function in liver [7]. In islets from healthy rats, imeglimin was also shown to amplify insulin secretion in response to obligate mitochondrial fuels [8]. Here, we showed that imeglimin increased islet ATP levels, an effect that may be consistent with the potential to enhance mitochondrial metabolism. The lack of diazoxide inhibition of Imeglimin’s effect is still compatible with enhanced mitochondrial function since it is well known that additional anaplerotic mitochondrial metabolic cycles also mediate GSIS without requiring downstream K^+^-ATP channel closure [41].

Given its known roles in mitochondrial function, we measured NAD^+^ and demonstrated an increase with imeglimin, and with nicotinamide, a substrate for NAD^+^ production. Importantly, exogenous nicotinamide was previously shown to enhance GSIS in rodent and human islets [42–44]. We confirmed this effect and showed that providing additional substrate for NAD^+^ synthesis–low nicotinamide concentrations–appeared to act in concert with imeglimin to augment GSIS. These results suggest that pathways emanating from NAD^+^ remain competent in GK islets and may be involved in mediating imeglimin’s efficacy. NAD^+^ biogenesis occurs via *de novo* synthesis from tryptophan or the salvage pathway from nicotinamide via NAMPT [16, 45]. Gallotannin, which inhibits NAD^+^ synthesis via both pathways [17, 18], was used to provide further results suggesting that imeglimin’s effect to increase the NAD^+^ pool involves new synthesis of NAD^+^. We also excluded a direct effect of imeglimin on NAMPT activity *in vitro*. The effect of imeglimin to induce NAMPT gene expression and activity is intriguing but it is uncertain if this fully accounts for the net increase in NAD^+^ given the short time frame within which these effects were seen. Relevance of the potential role of NAMPT is underscored by studies showing NAMPT expression in β-cells (including human) and that NAMPT haplodeficiency impairs GSIS in mice [19, 46].

Increased intracellular Ca^2+^ is critical for insulin granule exocytosis; Ca^2+^ sources include both extracellular (via voltage-gated channels in response to K^+^-ATP closure) and intracellular pools [31, 47, 48]. Having observed that imeglimin can augment Ca^2+^ mobilization, we assessed a potential link to NAD^+^ generation. In addition to other roles [45, 49], metabolism of NAD^+^ by CD38 generates key second messengers–cADPR and NAADP—that are implicated in Ca^2+^ signaling [45, 50]. Increases in cADPR, in turn, can activate ryanodine receptors resulting in mobilization of Ca^2+^ stores from ER [23, 48, 51] and this pathway is reportedly operative in pancreatic β-cells [50, 52]. Our results suggest that imeglimin ‘s mechanism is dependent on components of this pathway. However, the efficiency of CD38 knockdown was limited and additional studies will be required to confirm and extend these findings. In particular, future studies would benefit from also including a genetic knockout rodent model(s) such as that described by Kato et al. [53]. Additional support for the potential role of cADPR and/or NAADP in imeglimin’s actions could also be derived from the use of specific antagonists that have been developed as tools, such as 8-Br-cADPR [54]. Although CD38 is described as an ectoenzyme [17], it also exists in an inward orientation and can consume intracellular NAD^+^ [17, 55]. This pathway is highlighted by increases in islet cADPR and GSIS resulting from β-cell-specific CD38 overexpression in mice [56]. However, we acknowledge cADPR’s role in islet function is controversial; especially given an inability to consistently show that cADPR drives Ca^2+^ release [57, 58]. Some of these discrepancies may have resulted from differences in species and methodologies [50].

In assessing the potential role of an NAD^+^ mediated effect to enhance Ca^2+^ mobilization, our experiments were limited by an inability to measure levels of cADPR in islets from this diabetic rat model, not further interrogating the possible role of NAADP or showing a direct correlation between changes in Ca^2+^ and the apparent effects of modulating CD38 or RyR. Our studies were also restricted to short time points and may have missed additional, later, effects. There is also a clear need to more precisely define a direct molecular target(s) for imeglimin including mechanism(s) that may be responsible for induction of NAMPT gene expression.

**Fig 7:**
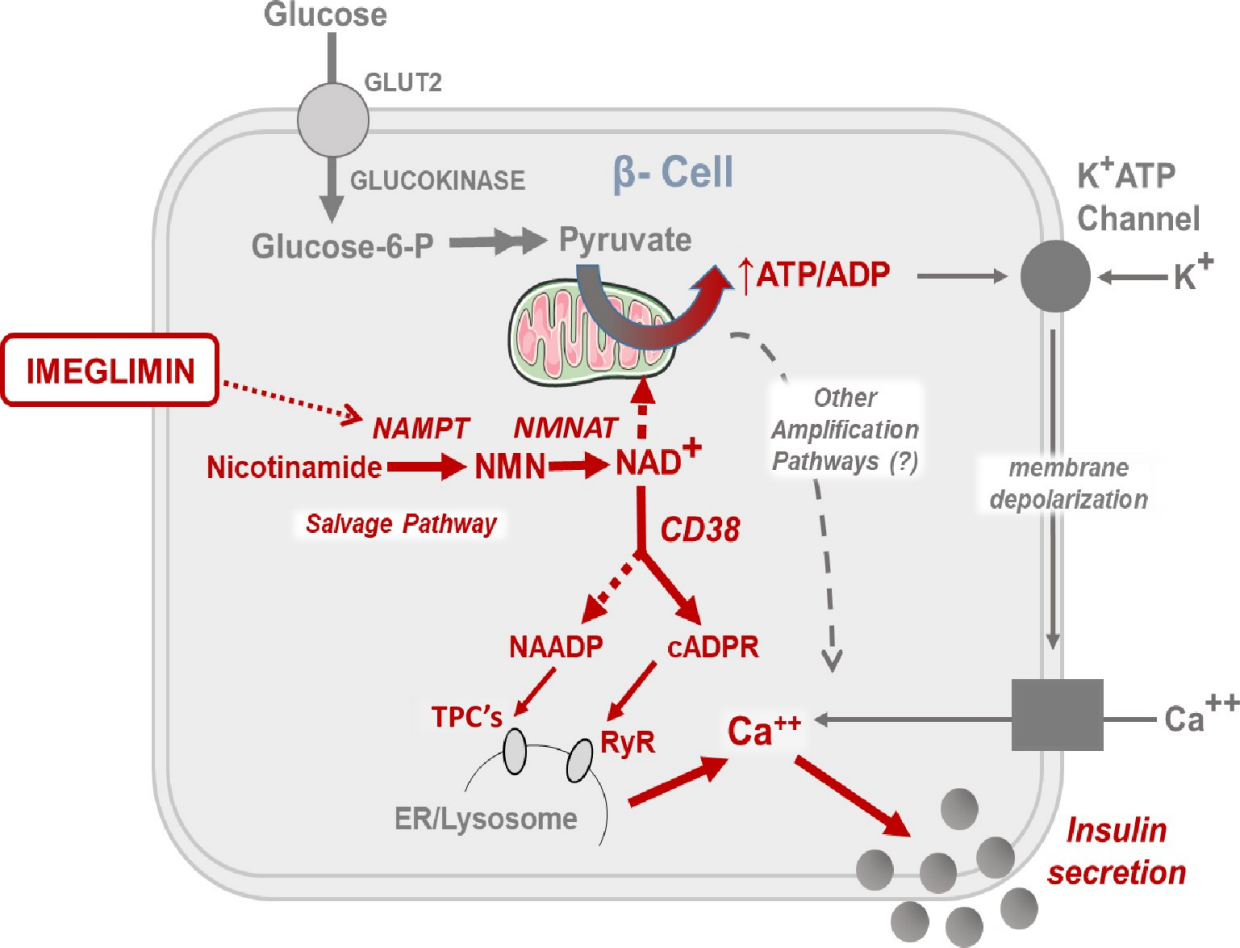
Proposed model for mechanism of imeglimin action in islet β-cells. The effects of imeglimin in the context of glucose stimulation are highlighted in red (text and arrows).

In summary, we have demonstrated an effect of imeglimin to acutely and directly restore GSIS in diseased islets from a rat model that closely resembles human Type 2 diabetes. Importantly, mechanisms employed by other classes of antidiabetic medications including incretin mimetics, sulphonylureas, and metformin were excluded. The results described here are also consistent with a potential proposed mode of action (Fig 7) that involves a pathway leading to increased NAD^+^ content which has been implicated in the regulation of intracellular Ca^2+^. This pathway is quite distinct and does not appear to overlap with mechanisms employed with other classes of antidiabetic therapies. Additional studies will be required to assess the extent to which pathways implicated in the present studies are also modulated by imeglimin in human islets. Although prior literature has shown that a predominant effect of imeglimin in animals and humans involves amplification of GSIS, the aforementioned findings from the current experiments are novel and not previously described. The results reported here are also consistent with existing clinical data where imeglimin has been shown to effectively treat hyperglycemia without any additional risk of hypoglycemia.

## Supporting information

S1 Fig Imeglimin effects on insulin release from GK rat islets in low vs. high glucose conditions. (PDF)

S2 Fig Comparison of GSIS in isolated islets from healthy wistar vs. diabetic GK rats. (PDF)

S3 Fig Effect of imeglimin on GSIS in isolated islets from a patient donor with type 2 diabetes. (PDF)

S4 Fig Effects of imeglimin on GSIS in GK rat islets when added to maximal GLP1. (PDF)

S5 Fig Inhibition of phospholipase C signaling. (PDF)

S6 Fig Metformin does not affect ATP levels in isolated GK rat islets. (PDF)

S7 Fig Increases in NAD^+^ content of GK rat islets are sufficient to augment insulin release. (PDF)

S8 Fig Imeglimin does not modulate the activity of recombinant NAMPT. (PDF)

S9 Fig Control experiments with diazoxide, sulphonylureas, KCl. (PDF)

S10 Fig Comparison of intracellular Ca2+ responses to glucose in wistar vs. GK rat islets. (PDF)

S11 Fig Lack of effect of imeglimin on intracellular Ca2+ in the presence of low glucose. (PDF)

S12 Fig CD38 gene expression after siRNA knockdown in primary cultured GK rat islets. (PDF)

S1 Table Summary of additional experiments demonstrating increased GSIS with imeglimin in GK rat islets. (PDF)
