# Supplementary material for: Imeglimin amplifies glucose-stimulated insulin release from diabetic islets via a distinct mechanism of action
Source: PLoS One. 2021 Feb 19;16(2):e0241651. doi: 10.1371/journal.pone.0241651 (PMC7894908; doi:10.1371/journal.pone.0241651)
Supplement: S1 Fig — (PDF) [file pone.0241651.s001.pdf]

S1 Fig. Imeglimin Effects on Insulin Release from GK Rat Islets in Low vs. High Glucose Conditions

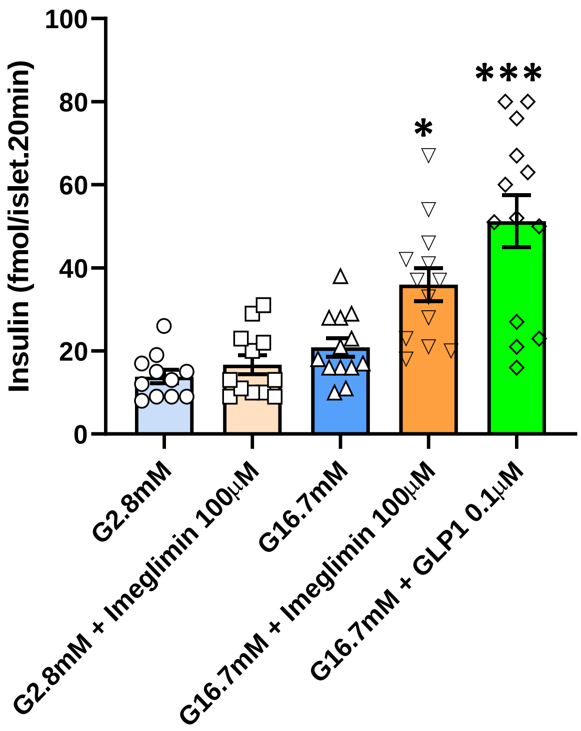

Islets isolated from GK rats were incubated (20 min.) in the presence of glucose 2.8 mM or 16.7 mM, with or without 100 μM Imeglimin or GLP1. The insulin levels were measured in supernatants using an Elisa test. Mean + SEM values are shown (n=12-13 wells with 10 islets/well for each condition). In the presence of high glucose, Imeglimin potentiated insulin secretion (+72%, *p<0.05); the effect of GLP-1 was also significant (***p<0.001); no effect of Imeglimin was measurable in low glucose conditions.
