# Supplementary material for: Imeglimin amplifies glucose-stimulated insulin release from diabetic islets via a distinct mechanism of action
Source: PLoS One. 2021 Feb 19;16(2):e0241651. doi: 10.1371/journal.pone.0241651 (PMC7894908; doi:10.1371/journal.pone.0241651)
Supplement: S2 Fig — (PDF) [file pone.0241651.s002.pdf]

S2 Fig. Comparison of GSIS in Isolated Islets from Healthy Wistar vs. Diabetic GK Rats

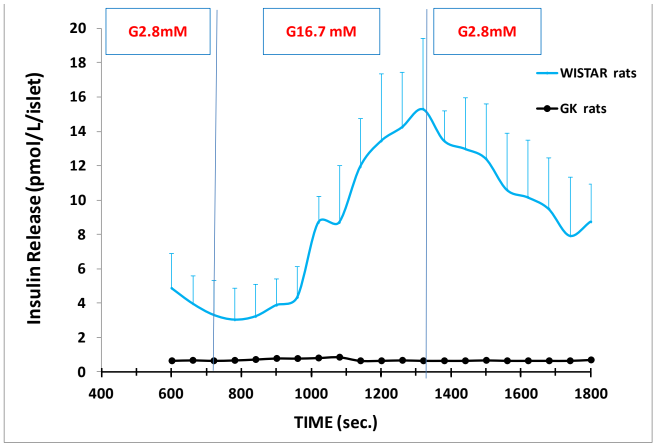

Islets from GK (black curve) and Wistar (blue curve) rats were alternately perifused with glucose 2.8 mM and 16.7 mM. The insulin level was measured in perifusate every min from 0 sec. to 1800 sec. Data were derived from 4 independent experiments.
