# Supplementary material for: Imeglimin amplifies glucose-stimulated insulin release from diabetic islets via a distinct mechanism of action
Source: PLoS One. 2021 Feb 19;16(2):e0241651. doi: 10.1371/journal.pone.0241651 (PMC7894908; doi:10.1371/journal.pone.0241651)
Supplement: S3 Fig — (PDF) [file pone.0241651.s003.pdf]

S3 Fig. Effect of Imeglimin on GSIS in Isolated Islets from a Patient Donor with Type 2 Diabetes

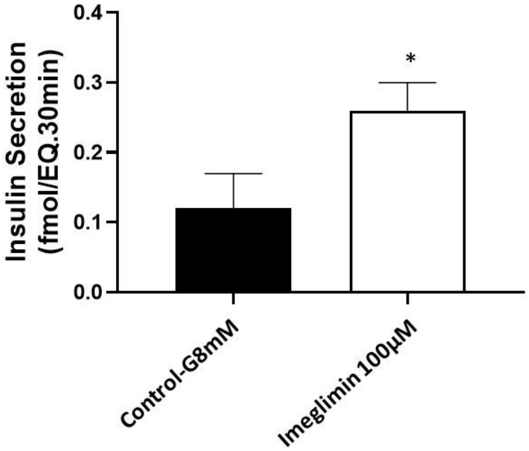

Imeglimin effect on insulin secretion (30min. static incubation) was tested in human islets obtained from a patient with type 2 diabetes. Imeglimin (100 µM) in presence of glucose 8 mM (G8mM) significantly increased insulin secretion (+129%, $p<0.05$ vs. Control-G 8 mM). Mean + SEM of 8-10 observations shown.
