# Supplementary material for: Imeglimin amplifies glucose-stimulated insulin release from diabetic islets via a distinct mechanism of action
Source: PLoS One. 2021 Feb 19;16(2):e0241651. doi: 10.1371/journal.pone.0241651 (PMC7894908; doi:10.1371/journal.pone.0241651)
Supplement: S4 Fig — (PDF) [file pone.0241651.s004.pdf]

S4 Fig. Effects of Imeglimin on GSIS in GK Rat Islets when Added to Maximal GLP1

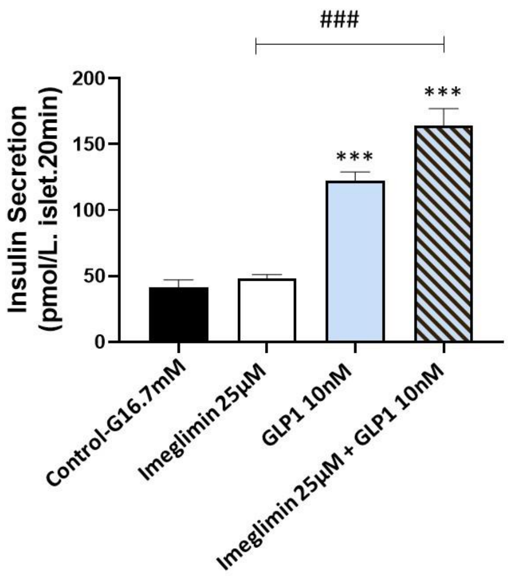

GLP1 alone was tested at 10 nM; a 299% (maximal) increase in insulin secretion was observed ($p<0.001$). The addition of a submaximal Imeglimin concentration (25 µM) to GLP1 resulted in a (+34%, NS) trend towards further insulin release. Mean ± SEM of 10-11 observations per group; ***$p<0.001$ vs. control G16.7mM ; ### $p<0.001$ vs. Imeglimin alone.
