# Supplementary material for: Imeglimin amplifies glucose-stimulated insulin release from diabetic islets via a distinct mechanism of action
Source: PLoS One. 2021 Feb 19;16(2):e0241651. doi: 10.1371/journal.pone.0241651 (PMC7894908; doi:10.1371/journal.pone.0241651)
Supplement: S5 Fig — (PDF) [file pone.0241651.s005.pdf]

S5 Fig. Inhibition of Phospholipase C Signaling

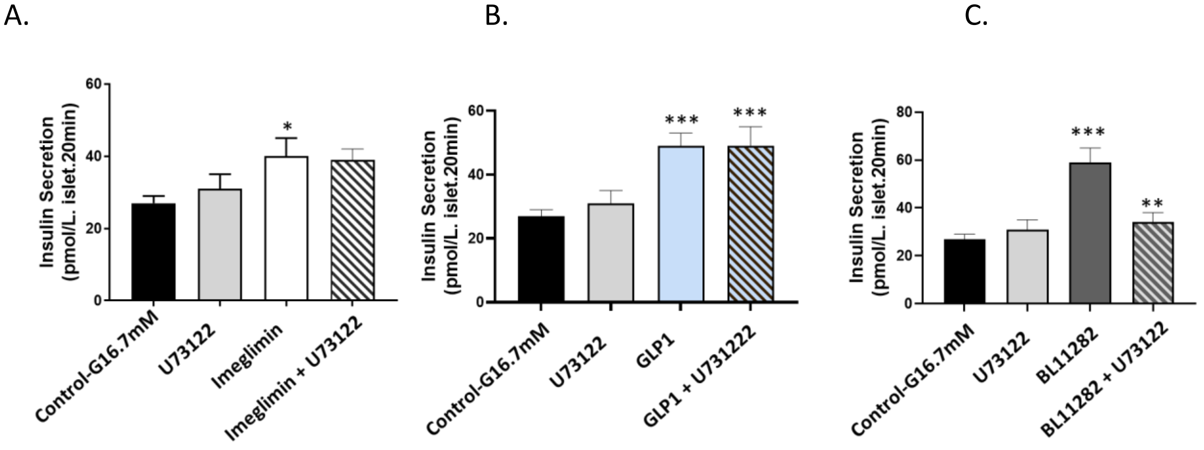

A. Static incubation (20 min.) of GK rat islets in high glucose with Imeglimin (100µM) produced the expected increase in insulin release (+47%, *p<0.05, n=14). Co-incubation with U73122 (10µM) did not modify Imeglimin's effect (+43%) vs. Imeglimin alone. U73122 alone also had no effect on glucose-stimulated insulin secretion.

B. GSIS was significantly (***, p<0.001) amplified by incubation with GLP1 (0.1 µM) with no effect of U73122.

C. In contrast, glucose-stimulated insulin release (+117%, *** p<0.001, n=10) in response to a PLC pathway positive control (BL11282, 10µM) was effectively inhibited by U73122 (-63% vs. BL11282 alone, ** p<0.01, n=10).
