# Supplementary material for: Imeglimin amplifies glucose-stimulated insulin release from diabetic islets via a distinct mechanism of action
Source: PLoS One. 2021 Feb 19;16(2):e0241651. doi: 10.1371/journal.pone.0241651 (PMC7894908; doi:10.1371/journal.pone.0241651)
Supplement: S6 Fig — (PDF) [file pone.0241651.s006.pdf]

S6 Fig. Metformin Does Not Affect ATP Levels in Isolated GK Rat Islets

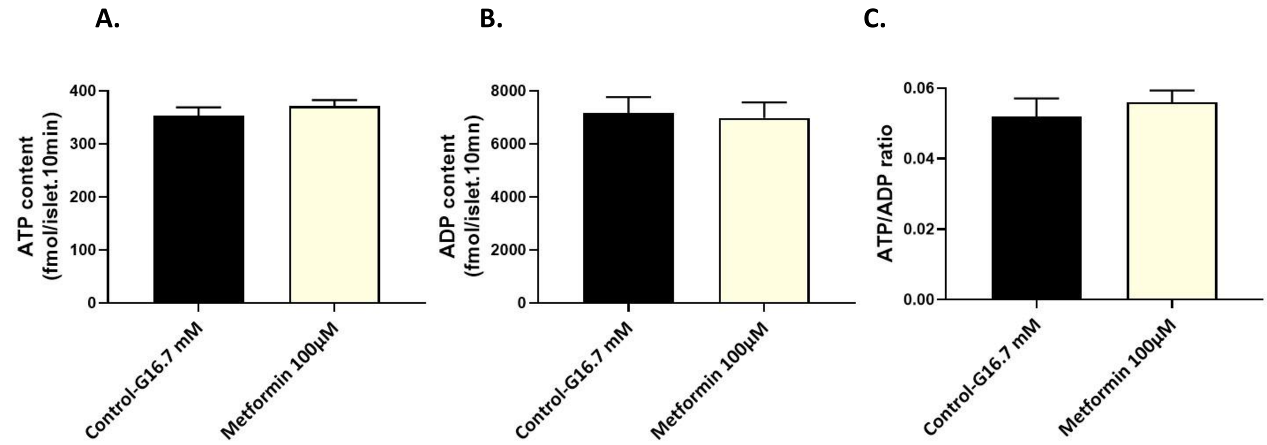

Metformin (100 μM) was added to GK rat islets in the presence of high (16.7 mM) glucose. No effect of metformin on ATP (A), ADP (B) or ATP/ADP (C) ratio was detected. Mean ± SEM values are shown (n=10).
