# Supplementary material for: Imeglimin amplifies glucose-stimulated insulin release from diabetic islets via a distinct mechanism of action
Source: PLoS One. 2021 Feb 19;16(2):e0241651. doi: 10.1371/journal.pone.0241651 (PMC7894908; doi:10.1371/journal.pone.0241651)
Supplement: S7 Fig — (PDF) [file pone.0241651.s007.pdf]

S7 Fig. Increases in NAD^+ Content of GK Rat Islets are Sufficient to Augment Insulin Release

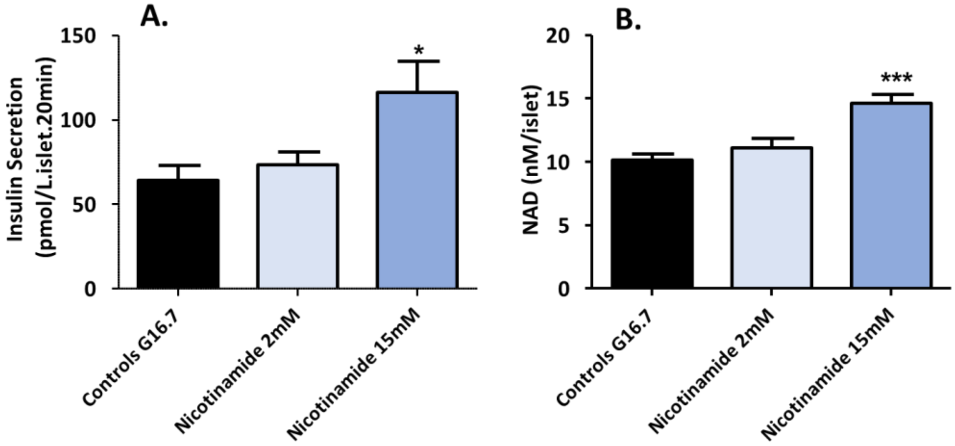

Islets from GK rats were incubated for 20 min. in the presence of 16.7 mM glucose with or without Nicotinamide (2 mM and 15 mM). Insulin concentrations in supernatant (A) and NAD^+ content in islets (B) were subsequently measured; mean (n=10) $\pm$ SEM values are shown; *$p<0.05$; ***$p<0.001$ vs. controls.
