# Supplementary material for: Imeglimin amplifies glucose-stimulated insulin release from diabetic islets via a distinct mechanism of action
Source: PLoS One. 2021 Feb 19;16(2):e0241651. doi: 10.1371/journal.pone.0241651 (PMC7894908; doi:10.1371/journal.pone.0241651)
Supplement: S8 Fig — (PDF) [file pone.0241651.s008.pdf]

S8 Fig. Imeglimin Does Not Modulate the Activity of Recombinant NAMPT

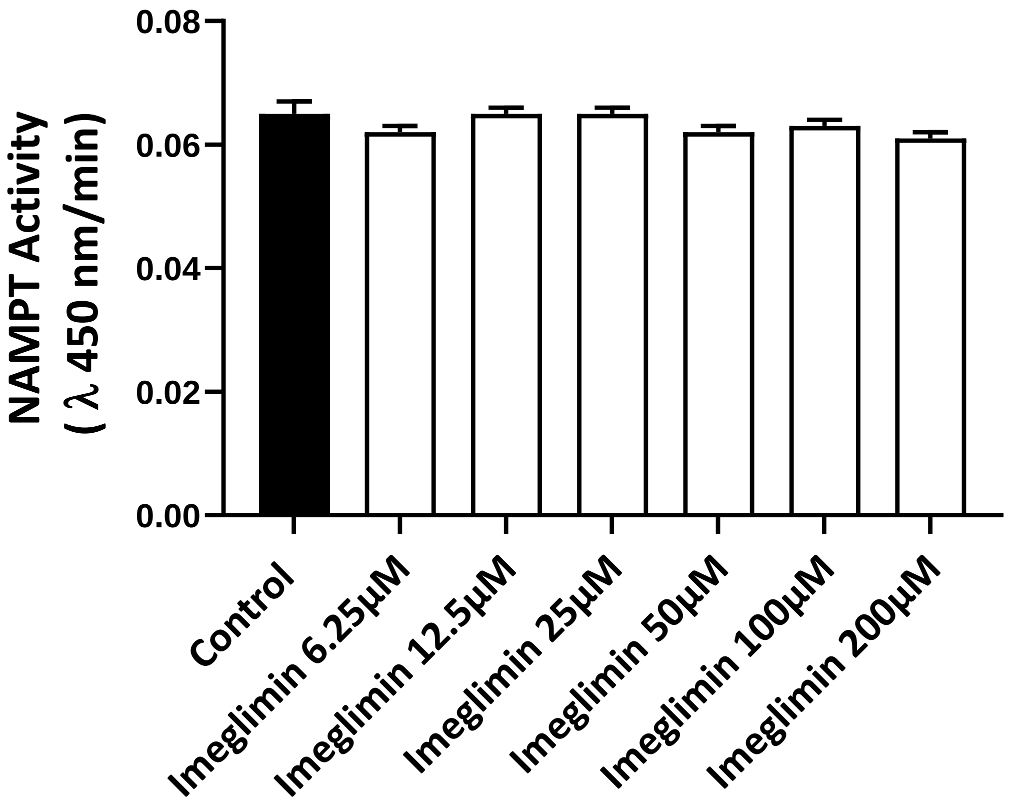

Isolated recombinant NAMPT enzyme activity was not altered by Imeglimin at 6 different concentrations ranging from 6.25 to 200 µM; mean (± SEM) activity vs. control ranged from 94-101%, all not significant (n=3-8 per group).
