# Supplementary material for: Imeglimin amplifies glucose-stimulated insulin release from diabetic islets via a distinct mechanism of action
Source: PLoS One. 2021 Feb 19;16(2):e0241651. doi: 10.1371/journal.pone.0241651 (PMC7894908; doi:10.1371/journal.pone.0241651)
Supplement: S9 Fig — (PDF) [file pone.0241651.s009.pdf]

S9 Fig. Control Experiments with Diazoxide, Sulphonylureas, KCl

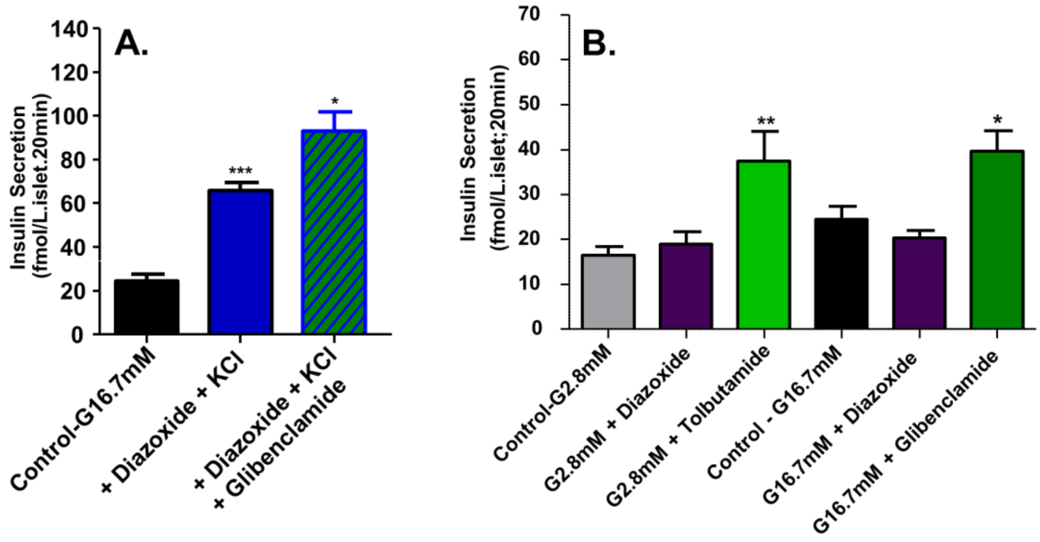

A. Islets from GK rats were treated (20 min) with KCl (30 mM) and Diazoxide (400µM) in the presence or absence of Glibenclamide 40µM (Mean ± SEM of 8 to 10 observations per group). B. Islets from GK rats were incubated (20 min.) in the presence of Diazoxide or Tolbutamide (500 µM) at 2.8 mM glucose and Diazoxide (400µM) and Glibenclamide (40µM) at 16.7mM glucose (Mean ± SEM of 4 to 11 observations per group). $*p<0.05$, $**p<0.01$, $***p<0.001$ vs. respective control value.
