# Supplementary material for: Imeglimin amplifies glucose-stimulated insulin release from diabetic islets via a distinct mechanism of action
Source: PLoS One. 2021 Feb 19;16(2):e0241651. doi: 10.1371/journal.pone.0241651 (PMC7894908; doi:10.1371/journal.pone.0241651)
Supplement: S10 Fig — (PDF) [file pone.0241651.s010.pdf]

S10 Fig. Comparison of Intracellular Ca2+ Responses to Glucose in Wistar vs. GK Rat Islets

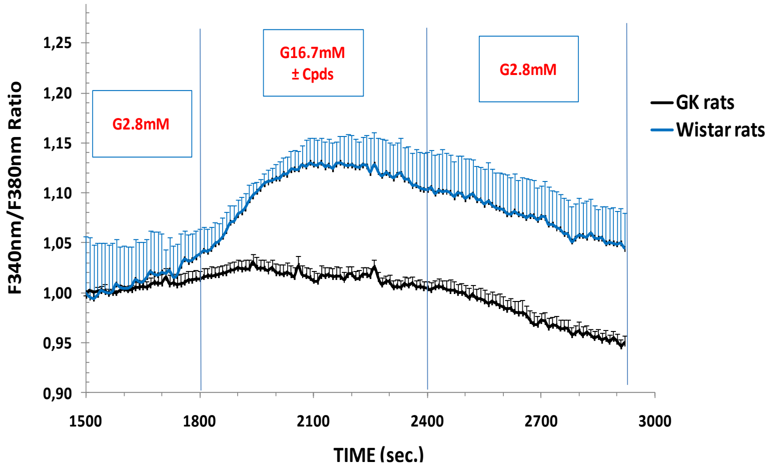

Islets from Wistar and GK rats were perifused in the presence of glucose 2.8 and 16.7 mM. The intracellular calcium levels were measured using individual islets by successive excitation at 340 nm and 380 nm, and the fluorescence emitted at 510 nm was measured every 10 sec. from 1200 sec. to 2920 sec. Data are derived from 8 experiments per group.
