# Supplementary material for: Imeglimin amplifies glucose-stimulated insulin release from diabetic islets via a distinct mechanism of action
Source: PLoS One. 2021 Feb 19;16(2):e0241651. doi: 10.1371/journal.pone.0241651 (PMC7894908; doi:10.1371/journal.pone.0241651)
Supplement: S11 Fig — (PDF) [file pone.0241651.s011.pdf]

S11 Fig. Lack of Effect of Imeglimin on Intracellular Ca2+ in the Presence of Low Glucose

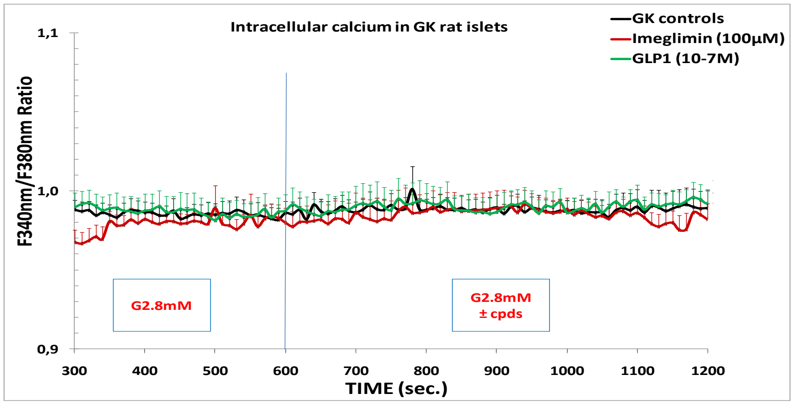

Islets from GK rats were perifused successively with glucose 2.8 mM for 600 seconds and a further 600 seconds with G2.8 mM for control (black curve) or G2.8 mM + Imeglimin 100µM (red curve) or G2.8 mM + GLP1 0.1µM (green curve). The intracellular calcium levels were measured using individual islets by successive excitation at 340 nm and 380 nm, and the fluorescence emitted at 510 nm was measured every 10 sec. Data are derived from 8 experiments per group.
