# Supplementary material for: Imeglimin amplifies glucose-stimulated insulin release from diabetic islets via a distinct mechanism of action
Source: PLoS One. 2021 Feb 19;16(2):e0241651. doi: 10.1371/journal.pone.0241651 (PMC7894908; doi:10.1371/journal.pone.0241651)
Supplement: S12 Fig — (PDF) [file pone.0241651.s012.pdf]

S12 Fig. CD38 Gene Expression after siRNA Knockdown in Primary Cultured GK Rat Islets

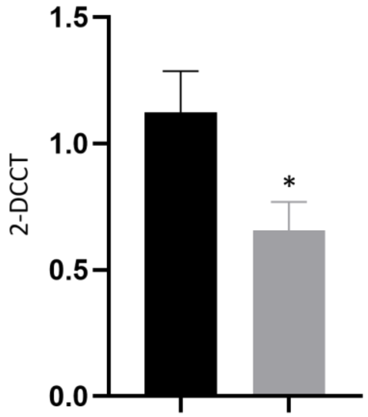

Example experiment where permeabilized islets were treated with scrambled sequence Control (black bar) siRNA or siRNA targeted to CD38 (grey bar) and then cultured for 48h before CD38 mRNA levels were determined as described in Methods. Mean ± SEM of 17 observations per group; *p<0.05 vs. Control.
