# Supplementary material for: Imeglimin amplifies glucose-stimulated insulin release from diabetic islets via a distinct mechanism of action
Source: PLoS One. 2021 Feb 19;16(2):e0241651. doi: 10.1371/journal.pone.0241651 (PMC7894908; doi:10.1371/journal.pone.0241651)
Supplement: S1 Table — (PDF) [file pone.0241651.s013.pdf]

S1 Table Summary of Additional Experiments
Demonstrating Increased GSIS with Imeglimin in GK Rat Islets

Experiment	Mean Insulin Secretion (% of High Glucose Control)	Number of Observations	p Value	Comment
1.	152 ± 13	13	<0.05	Data shown in Table II
2.	172 ± 19	13	<0.05	Data shown in Fig. S1
3.	157 ± 13	10	<0.05	Data shown in Fig. 5B
4.	147 ± 18	14	<0.05	-
5.	140 ± 5	7	<0.01	30 min.
6.	166 ± 18	14	<0.05	30 min.

In addition to the primary datasets described in main Figures 2B, 2D, several additional static incubation experiments captured the effect of Imeglimin (100 µM in each study) to enhance GSIS in islets isolated from GK rats. Except where noted, islets were incubated 20 min. in the presence of high (16.7 mM) glucose with or without Imeglimin. Data are expressed as the mean percentage (%) ± SEM of the mean high glucose control from the respective study.
